# Prevalence and Prognostic Value of Pulmonary Hypertension in Chronic Obstructive Pulmonary Disease

**DOI:** 10.7759/cureus.78769

**Published:** 2025-02-09

**Authors:** Evgeni V Mekov, Nikolay A Yanev, Nedelina Kurtelova, Teodora Mihalova, Adelina Tsakova, Yordanka Yamakova, Marc Miravitlles, Rosen E Petkov

**Affiliations:** 1 Department of Pulmonary Diseases, Medical University - Sofia, Sofia, BGR; 2 Department of Respiratory Diseases, Medical University - Sofia, Sofia, BGR; 3 Central Clinical Laboratory, Medical University - Sofia, Sofia, BGR; 4 Department of Anesthesiology and Intensive Care, Medical University - Sofia, Sofia, BGR; 5 Department of Pneumology, Hospital Universitari Vall d’Hebron/Vall d’Hebron Institut de Recerca (VHIR) Vall d’Hebron Hospital Campus, CIBER de Enfermedades Respiratorias (CIBERES), Barcelona, ESP

**Keywords:** comorbidities, copd, echocardiography, prevalence, pulmonary hypertension

## Abstract

Introduction

Pulmonary hypertension (PH) significantly influences chronic obstructive pulmonary disease (COPD) outcomes by exacerbating symptoms, increasing the frequency and severity of exacerbations, and contributing to higher hospitalization rates and mortality. Ultrasound assessment of mean pulmonary arterial pressure (PAPm) may contribute to the severity assessment of COPD. This study aims to assess the one-year prognostic value of PAPm at rest and after exercise in COPD patients.

Methods

This was an observational, prospective study of stable COPD patients over 40 years of age who were current or former smokers with a smoking history of >10 pack-years and a postbronchodilator FEV1 (forced expiratory volume in 1 second)/FVC (forced vital capacity) <0.7. Exclusion criteria were other significant lung diseases, systemic inflammatory diseases, or non-compliance with study procedures. PAPm was calculated using peak tricuspid regurgitation velocity via transthoracic echocardiography at rest and after a 6-minute walking test (6MWT) as a predictor of COPD exacerbations and death in one year. Patients were followed for 12 months to assess COPD exacerbations and mortality outcomes.

Results

A total of 96 patients were analyzed with a mean FEV1 (% predicted) of 55.8%. The prevalence of PH in this group was 52.1% (50/96). PAPm at rest was a stronger predictor of exacerbations, with an area under the curve (AUC) of 0.732, compared to PAPm after exercise (AUC: 0.700). The patients with PH had a significantly higher number of exacerbations (1.65 vs 0.89, p = 0.002). The patients with PAPm ≥30 after exercise also have a considerably higher number of exacerbations (1.64 vs. 1.1, p = 0.026). In univariate analysis, age, pack-years, FEV1, FVC, 6-minute walk distance (6MWD), COPD Assessment Test (CAT) score, previous exacerbations, PAPm, and PAPm after exertion are significant predictors for exacerbations and/or composite outcome (exacerbation or death). In multivariate analysis, however, only previous exacerbations remain significant in all models.

Conclusion

This study found a 52.1% prevalence of PH. Patients with PH at rest and after exercise had more frequent exacerbations during follow-up. Integrating non-invasive PAPm measurement into routine clinical practice could enhance risk stratification, guide treatment strategies, and improve patient outcomes.

## Introduction

Pulmonary hypertension (PH) is a significant and underrecognized complication in chronic obstructive pulmonary disease (COPD). While PH affects approximately 1% of the global population [1], its prevalence rises considerably among COPD patients, reflecting the close relationship between lung diseases and pulmonary vascular dysfunction [2]. PH is defined in hemodynamic terms by an abnormal increase in mean pulmonary arterial pressure (PAPm). According to the latest definition, the proposed cutoff for PH is PAPm >20 mmHg [3].

Lung disease, especially COPD, is the second most common cause of PH [4]. PH is a frequent and important complication of COPD and may be associated with a worse clinical course, frequent exacerbations, shorter survival, and higher healthcare resource utilization [5].

This study aims to evaluate the one-year prognostic value of PAPm at rest and after exercise as predictors of COPD exacerbations, with a focus on their potential integration into routine clinical care.

## Materials and methods

This was an observational and prospective study. Outpatients attending the COPD clinic of a tertiary hospital were invited to participate between 01.01.2018 and 01.07.2018. During the inclusion visit, demographic characteristics, such as age and sex, and clinical characteristics of the disease, such as post-bronchodilator spirometry and the number of exacerbations in the previous year, were collected. Shortness of breath was assessed with the modified Medical Research Council (mMRC) dyspnea scale and quality of life with the COPD Assessment Test (CAT) questionnaire. Patients with two or more moderate exacerbations or one or more severe exacerbations (hospitalization) during the previous year were considered “frequent exacerbators” (GOLD group E).

Patients over 40 years of age, current or former smokers with more than 10 pack-years, and diagnosed with COPD with a postbronchodilator FEV1 (forced expiratory volume in 1 second)/FVC (forced vital capacity) <0.7 were included. They had to be in stable condition for at least a month. Exclusion criteria were the presence of other lung diseases (cystic fibrosis, severe bronchiectasis, cancer, or restrictive lung disease), systemic inflammatory disease, and lack of compliance with the procedures (e.g. quality-of-life questionnaires, 6-minute walking test (6MWT)).

For assessment of PH and PAPm, transthoracic echocardiography (echoCG) was performed. A sector transducer with a frequency of 2-3.5 MHz was used in 2D and M-Mode. PAPm was calculated using peak tricuspid regurgitation velocity. Afterward, the patients performed a 6MWT according to the American Thoracic Society guidelines [6] and the echoCG was repeated right after the test. The ultrasound examinations and measurements were performed by one trained specialist (RP) who is expert-level echoCG certified with more than 20 years of experience.

Patients were followed for 12 months with clinical visits scheduled at six and 12 months, during which information was collected about the number and severity of exacerbations during that period. The one-year outcomes were as follows:

- Composite endpoint consisting of moderate or severe exacerbation or death of any cause (yes/no); frequent exacerbator (yes/no).

- The number of exacerbations (number/year).

Moderate exacerbation was defined as an acute increase in respiratory symptoms requiring ambulatory treatment with antibiotics and/or systemic corticosteroids. When the episode required treatment in a hospital setting or assistance in the emergency room for at least 24 hours, it was considered severe [7].

The study was approved by the local ethics committee and all the participants provided signed informed consent and received the usual treatment schedule according to the criteria of the attending physician.

The methodology and the cohort in this study are the same as those in our previous research focusing on ultrasound results [8]. The present study focuses on PH in these COPD patients.

Statistical analysis

An exploratory analysis was performed on the impact of PAPm at rest and after exertion on outcomes. All results are expressed as mean ± SD or percentages when appropriate.

Patients were divided into two groups according to the PAPm cutoff of 20 mmHg into PH and non-PH. Assumptions of normality and homogeneity of variances were tested for all parametric analyses. Quantitative variables between groups with normal distribution were compared with Student’s t-test. For non-normally distributed data, the Mann-Whitney U test was used. Qualitative variables were described with frequencies and proportions and were compared by the chi-squared test. Kaplan-Meier survival analysis was used for the time to the composite endpoint. Correlations were tested with the Pearson correlation coefficient.

Further analyses were performed by searching the optimal cutoff points for PAPm using the cutpointr package (https://cran.r-project.org/web/packages/cutpointr/vignettes/cutpointr.html) with a target to maximize the metric function “Youden’s J” (method = maximize_metric, metric = youden). Receiver operating characteristic (ROC) curves were used for performance evaluation of the parameters at rest and after 6MWT and AUC were calculated.

Univariate and multivariate analyses were performed for identifying the factors, associated with the outcome. Variables with a significance <0.2 in the univariate analysis were included as independent variables. A multivariate Cox model was developed using backward stepwise logistic regression analysis. The data were analyzed with RStudio v. 2022.02.3/R 4.2.2 (R Foundation for Statistical Computing, Vienna, Austria).

## Results

Population characteristics and prevalence of PH

A total of 96 patients with COPD were included. The mean age was 65.1 (standard deviation (SD): 8.1) years and the average FEV1 was 55.8% (SD: 18.3%). Table 1 summarizes the characteristics of the included patients. No patients were lost and three patients (3/96, 3.1%) died during the follow-up.

**Table 1:** Patient and disease characteristics. FEV1, forced expiratory volume in 1 second; FVC, forced vital capacity; CAT, COPD Assessment Test; COPD, chronic obstructive pulmonary disease; mMRC, modified Medical Research Council; 6MWT, 6-minute walking test; IQR, interquartile range; N/A, not applicable; PAPm, mean pulmonary arterial pressure; PH, pulmonary hypertension; SD, standard deviation. Note: Quantitative variables were compared using Student’s t-test. Qualitative variables were compared using the chi-squared test. *Values represent mean ± SD.

Characteristic	All patients (n = 96)	Patients without PH (n = 46)	Patients with PH (n = 50)	p-Value for PH vs no PH
Age (years)*	65.1 ± 8.1	62.5 ± 8.6	67.4 ± 6.9	0.003
Male, n (%)	60 (62.5%)	26 (56.5%)	34 (68%)	0.34
Active smokers, n (%)	33 (34.4%)	13 (28.3%)	20 (40%)	0.32
Smoking pack-years*	28.5 ± 14.9	24.2 ± 11.7	32.8 ± 15.7	0.003
FEV1 (%)*	55.8 ± 18.3	63.7 ± 16.1	48.4 ± 17.2	<0.0001
FVC (%)*	77.9 ± 22.5	85.9 ± 21.2	70.6 ± 21.2	0.0006
Distance at 6MWT (m)*	372 ± 110	428 ± 94	321 ± 99.5	<0.0001
CAT score*	15.6 ± 9.2	10.2 ± 6.9	20.6 ± 8.3	<0.0001
mMRC score:				<0.0001
- 0	11 (11.5%)	10 (21.7%)	1 (2%)	
- 1	15 (15.6%)	11 (23.9%)	4 (8%)	
- 2	28 (29.2%)	18 (39.1%)	10 (20%)	
- 3	41 (42.7%)	7 (15.2%)	34 (68%)	
- 4	1 (1%)	0	1 (2%)	
Moderate exacerbations in the previous year				0.064
- Mean ± SD	0.73 ± 0.84	0.57 ± 0.75	0.88 ± 0.90	
- Median (IQR)	1 (0-1)	0 (0-1)	1 (0-1)	
PAPm at rest (mmHg)*	22.4 ± 7.1	16.2 ± 3.5	28.1 ± 4.0	N/A
PAPm after 6MWT (mmHg)*	26.2 ± 8.6	19.5 ± 5.4	32.3 ± 5.9	N/A
Severe exacerbations in the previous year				0.274
- Mean ± SD	0.97 ± 0.87	0.93 ± 0.88	1.14 ± 0.95	
- Median (IQR)	1 (0-1)	1 (0-1)	1 (1-1.75)	
Frequent exacerbators in the previous year (GOLD E) (%)	73 (76%)	32 (70%)	41 (82%)	0.24

The prevalence of PH (PAPm at rest ≥20 mmHg) in this group is 52.1% (50/96). The patients with PH have significantly lower FEV1 (48.4% vs 63.7%, p < 0.0001) and FVC (70.6% vs 85.9%, p = 0.0006). PAPm correlates significantly with FEV1 (r = -0.45, p < 0.0001) and FVC (r = -0.34, p = 0.0006). PAPm at rest and after exercise also correlates with 6-minute walk distance (6MWD) (r = -0.58, p < 0.0001, and r = -0.52, p < 0.0001, respectively).

Outcomes for one-year follow-up

Sixty-four patients (67%) achieved the composite endpoint and 43 patients (45%) were frequent exacerbators (GOLD E) (Table 2).

**Table 2:** Main outcomes of the study. IQR: interquartile range; PH: pulmonary hypertension; SD: standard deviation. Note: Quantitative variables were compared using Student’s t-test. Qualitative variables were compared using the chi-squared test.

Outcome	All patients (n = 96)	Patients without PH at rest (n = 46)	Patients with PH (n = 50)	p-Value for PH vs no PH
Moderate exacerbations after 1 year				0.0004
- Mean (±SD)	0.86 ± 0.90	0.52 ± 0.76	1.16 ± 0.91	
- Median (IQR)	1 (0-1)	0 (0-1)	1 (1-1.75)	
Severe exacerbations after 1 year				0.358
- Mean ± SD	0.45 ± 0.87	0.36 ± 0.89	0.53 ± 0.84	
- Median (IQR)	0 (0-1)	0 (0-0)	0 (0-1)	
Total exacerbations	1.29 ± 1.21	0.89 ± 1.24	1.65 ± 1.07	0.002
Frequent exacerbators	43 (44.8%)	14 (30.4%)	29 (58%)	0.006
Death	3 (3.1%)	2 (4.3%)	1 (2.0%)	0.94
Composite endpoint	64 (66.7%)	22 (47.8%)	42 (84%)	0.0004

The patients with the composite endpoint had significantly higher PAPm at rest (24.3 vs 18.6 mmHg, p = 0.0001) and after exercise (28.2 vs 22.2 mmHg, p = 0.001).

The frequent exacerbators had a tendency for higher PAPm at rest (21.1 vs 23.9 mmHg, p = 0.051) and after exercise (27.8 vs 24.9 mmHg, p = 0.094).

The patients with PH at rest have a significantly higher number of exacerbations (1.65 vs. 0.89, p = 0.002). However, differences were only significant for moderate exacerbations (1.16 vs. 0.52, p = 0.0003), but not for severe exacerbations (0.53 vs. 0.36, p = 0.36). PAPm was a significant predictor only for moderate exacerbations (Table 3).

**Table 3:** Univariate and Cox multivariate analyses to determine factors associated with the number of moderate and severe exacerbations. FEV1, forced expiratory volume in 1 second; FVC, forced vital capacity; CAT, COPD Assessment Test; COPD, chronic obstructive pulmonary disease; 6MWT, 6-minute walking test; PAPm, mean pulmonary arterial pressure. *Factors showing an association in the univariate analyses (p < 0.2) were incorporated into the multivariate model. Final variable selection was performed using the backward stepwise selection method.

Variable	Moderate exacerbations				Severe exacerbations				Composite outcome			
	Univariate*		Multivariate		Univariate*		Multivariate		Univariate*		Multivariate	
	t-Value	p-Value	t-Value	p-Value	t-Value	p-Value	t-Value	p-Value	t-Value	p-Value	t-Value	p-Value
Age, years	1.11	0.268	-	-	0.54	0.593	-	-	2.29	0.024	-	-
Gender, male	-0.70	0.485	-	-	-0.67	0.5034	-	-	-0.889	0.376	-	-
Active smoker	-0.38	0.705	-	-	-0.25	0.801	-	-	-0.452	0.653	-	-
Smoking pack-years	3.03	0.0032	2.356	0.0207	-0.31	0.7588	-	-	2.26	0.027	-	-
FEV1, % predicted	-5.61	<0.0001	-	-	-1.97	0.0521	-	-	-5.81	<0.0001	-	-
FVC, % predicted	-6.10	<0.0001	-4.185	<0.0001	-1.25	0.2139	-	-	-6.36	<0.0001	-2.79	0.0065
Distance at 6MWT	-4.06	0.0001	-	-	-1.39	0.1692	-	-	-4.0	0.0001	-	-
CAT score	5.38	<0.0001	-	-	1.67	0.0975	-	-	6.48	<0.0001	1.71	0.091
Exacerbations in the previous year	3.61	0.0005	2.559	0.0122	4.42	<0.0001	3.66	0.0004	5.5	<0.0001	3.61	0.0005
PAPm	3.90	0.0002	1.492	0.1391	0.37	0.711	-	-	4.02	0.0001	-	-
PAPm after 6MWT	2.79	0.0065	-	-	0.61	0.543	-	-	3.44	0.0009	-	-

The patients with PAPm ≥30 after exercise have also significantly higher number of exacerbations (1.64 vs. 1.1, p = 0.026) and likewise, these differences were only significant for moderate exacerbations (1.21 vs. 0.67, p = 0.005), but not for severe exacerbations (0.48 vs. 0.43, p = 0.76).

PH and quality of life

**Figure 1:**
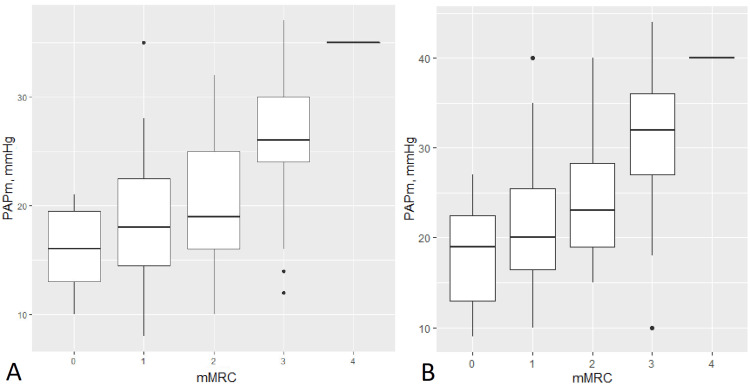
Mean PAPm at rest (A) and after 6MWT (B) according to mMRC group PAPm, pulmonary arterial pressure; 6MWT, 6-minute walking test; mMRC, modified Medical Research Council.

The mean CAT score was 15.6 points and the mMRC scores of 0, 1, 2, 3, and 4 have 11 (11.5%), 15 (15.6%), 28 (29.2%), 41 (42.7%), and 1 (1%) patients, respectively (Table 1). The patients with PH had a significantly worse quality of life (24.6 vs. 18.1 points on the CAT test, p < 0.0002). The dyspnea level (mMRC score) was correlated with PAPm (r = 0.54, p < 0.0001) (Figure 1). The presence of more symptoms (higher CAT score) was also correlated with PAPm (r = 0.61, p < 0.0001).

Factors associated with exacerbations: univariate and multivariate analyses

In univariate analysis age, pack-years, FEV1, FVC, 6MWD, CAT score, previous exacerbations, PAPm, and PAPm after exertion are significant predictors for exacerbations and/or composite outcome. In multivariate analysis, however, only previous exacerbations remain significant in all models. FVC is predictive for moderate exacerbations and composite outcome, and pack-years for moderate exacerbations.

Optimal cutoff values for PH at rest and after exercise

**Figure 2:**
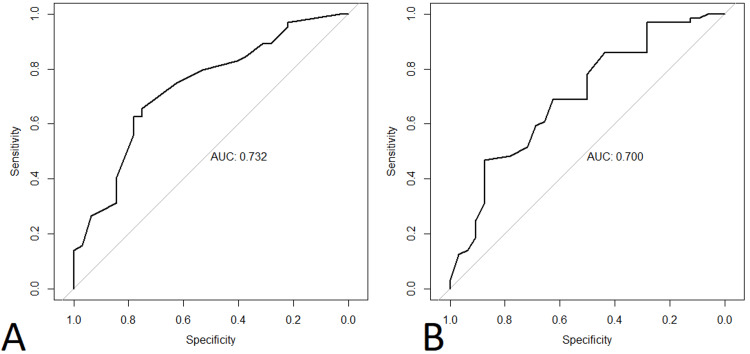
ROC curves for PAPm at rest (A) and after 6MWT (B) AUC, area under the curve; ROC, receiver operating characteristic; PAPm, mean pulmonary arterial pressure; 6MWT, 6-minute walking test.

**Figure 3:**
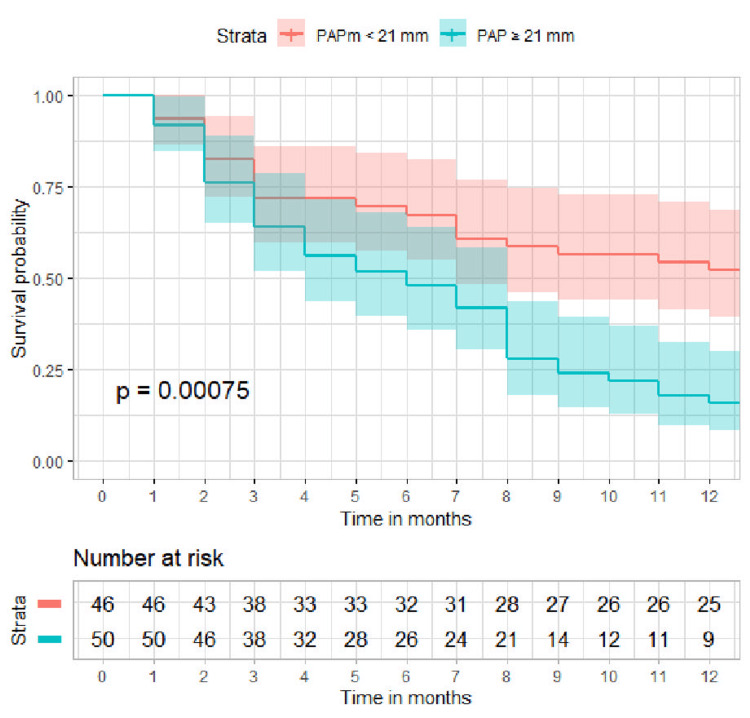
Survival curve for reaching the composite endpoint (time to exacerbation or death) stratified by PAPm at rest (≥21 vs. <21 mmHg) PAPm, mean pulmonary arterial pressure.

**Figure 4:**
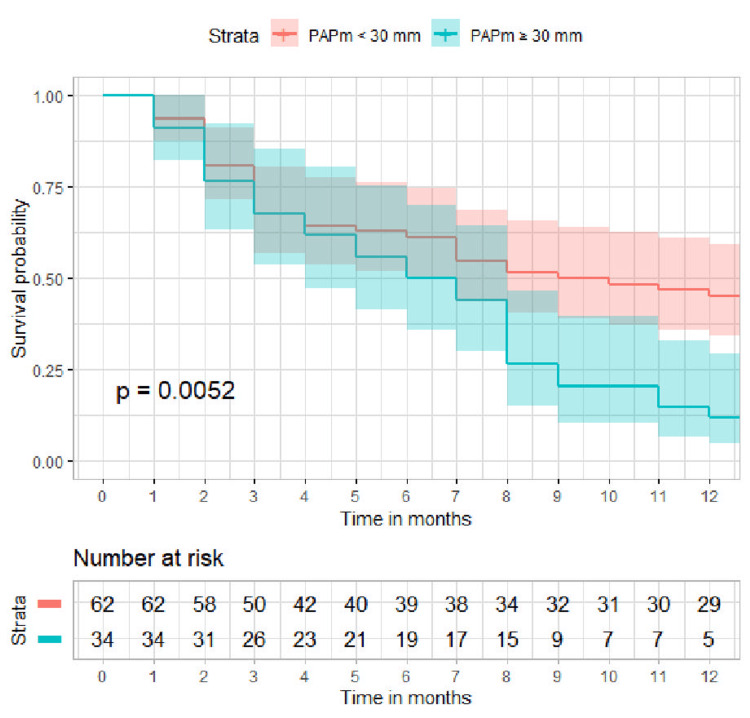
Survival curve for reaching the composite endpoint (time to exacerbation or death) stratified by PAPm after 6MWT (≥30 vs. <30 mmHg) PAPm, mean pulmonary arterial pressure; 6MWT, 6-minute walking test.

The optimal cutoff points that provide the maximum Youden’s J for the composite endpoint were 21 mmHg for PAPm at rest (AUC 0.732) and 30 mmHg after exertion (AUC 0.700), indicating good discrimination. ROC curves are shown in Figure 2. Patients with PAPm ≥21 mmHg were at increased risk for developing the composite endpoint (84% vs. 47.8% patients, p < 0.0001, Figure 3). Patients with PAPm ≥30 mmHg after 6MWT were also at increased risk for developing the composite endpoint (88.2% vs. 54.8% patients, p = 0.0002) with a specificity of 87.5% (Figure 4).

Regarding the exacerbation frequency, the optimal cutoff points that provide the maximum Youden’s J were the same -- 21 mmHg for PAPm at rest and 30 mmHg after exertion. Patients with PAPm ≥21 mmHg were at increased risk for frequent exacerbations (58.0% vs. 30.4%, p = 0.012). However, PAPm ≥30 mmHg after 6MWT is not a significant predictor for frequent exacerbations (54.8% vs. 40.0%, p = 0.25).

## Discussion

PH is a frequent complication of COPD, associated with increased exacerbation frequency, reduced survival, and greater healthcare resource utilization [5]. The prevalence of PH in patients with COPD is not negligible and depends on the severity of the disease. This study finds a 52.1% prevalence of PH. Up to 90% of patients might have abnormal PAPm (>20 mmHg), with most of them ranging between 20 and 35 mmHg [9,10]. In COPD, PH is usually of moderate severity, without altering right ventricular function in most cases [11,12].

Nevertheless, a small subgroup of patients may present severe PH, with PAP values exceeding those expected according to the severity of respiratory impairment. These patients may depict a clinical picture similar to more severe forms of PH and have greater mortality. Only 1-5% of patients with COPD have PAPm greater than 35-40 mmHg at rest [13], as in this study (1/96, 1%). Interestingly, even under moderate exercise conditions (e.g. 6MWT), most COPD patients show a rapid rise in PAPm, indicating loss of lung vasculature, vascular distensibility, and/or vessel recruitment capability [14]. It should be noted that in COPD, PH could be also due to comorbid left heart disease (more frequently), sleep-related breathing disorders, or chronic pulmonary thrombosis.

This study confirms the prognostic significance of PAPm in COPD patients. By identifying clinically relevant PAPm cutoffs, this research addresses the need for non-invasive, practical tools to improve risk stratification and guide personalized treatment strategies in COPD management. Moreover, the recently revised cutoff value for PH (>20 mmHg) is optimal in COPD patients. Other studies show that PAPm >20 mmHg is associated with increased mortality [15,16] and an increased risk for severe exacerbation [17,18]. However, in this study, PAPm is a better predictor for moderate exacerbations. Given the fact that PH is characteristic of severe COPD (i.e. decreased FEV1 and FVC), the predictive potential is probably diminished by the correlation with lung function. 

Remodeling of pulmonary vessels is the major cause of PH in COPD. It affects small and precapillary arteries and has been identified at different degrees of disease severity [19,20]. Other mechanisms such as a decrease in pulmonary vascular surface, parenchymal loss, air-trapping, abnormal pulmonary mechanics, and alveolar hypoxia could concur and promote the development of PH in COPD [2,21,22]. Hypoxemia has been suggested to be a PH trigger, but the relationship between PAPm and arterial pO_2_ (PaO_2_) has substantial variability [9], suggesting that there must be additional factors explaining the development of PH in COPD.

This study has several limitations that must be acknowledged. First, it was conducted at a single center, which may restrict the generalizability of the findings to broader COPD populations managed in different healthcare settings or with varying treatment practices. Additionally, the cohort size, although adequate for initial analyses, was relatively small. This limited sample size may reduce the statistical power to detect more subtle associations or subgroup differences, particularly in outcomes like severe exacerbations and mortality. Another limitation is the reliance on transthoracic EchoCG for the assessment of pulmonary arterial pressure. While EchoCG is a non-invasive and widely used method, its accuracy is inherently lower compared to invasive hemodynamic measurements, which could introduce variability in the results, and it cannot definitively differentiate between pre-capillary, post-capillary, and combined PH. Therefore, EchoCG alone cannot provide the cause for pulmonary hypertension, and most smokers will have left-sided heart disease, hypertension, or other comorbidities, which would affect treatment or management options. Additionally, EchoCG is a subjective assessment with inter-observer variability, which can affect the reliability and significance of the findings. Despite these limitations, the study provides valuable insights into the prognostic significance of PH in COPD and underscores the potential utility of non-invasive ultrasound assessments.

## Conclusions

This study highlights the high prevalence of PH in COPD patients, which is associated with increased exacerbation frequency and reduced quality of life. Non-invasive assessment of heart function by ultrasound measurement both at rest and after exercise could contribute to the assessment of disease severity and prognosis in COPD.
